# Sialic Acid Expression in the Mosquito *Aedes aegypti* and Its Possible Role in Dengue Virus-Vector Interactions

**DOI:** 10.1155/2015/504187

**Published:** 2015-03-22

**Authors:** Jorge Cime-Castillo, Philippe Delannoy, Guillermo Mendoza-Hernández, Verónica Monroy-Martínez, Anne Harduin-Lepers, Humberto Lanz-Mendoza, Fidel de la Cruz Hernández-Hernández, Edgar Zenteno, Carlos Cabello-Gutiérrez, Blanca H. Ruiz-Ordaz

**Affiliations:** ^1^Molecular Biology and Biotechnology Department, Biomedical Research Institute, National University of México (UNAM), 04510 México City, Mexico; ^2^Structural and Functional Glycobiology Unit, UMR 8576 CNRS, University of Sciences and Technologies of Lille, 59655 Villeneuve d'Ascq, France; ^3^Biochemistry Department, Faculty of Medicine, UNAM, 04510 México City, Mexico; ^4^CISEI, National Institute of Public Health, 62100 Cuernavaca, MOR, Mexico; ^5^Infectomics and Molecular Pathogenesis Department, CINVESTAV-IPN, 07360 México City, Mexico; ^6^Virology Department, National Respiratory Institute (INER), 14050 México City, Mexico

## Abstract

Dengue fever (DF) is the most prevalent arthropod-borne viral disease which affects humans. DF is caused by the four dengue virus (DENV) serotypes, which are transmitted to the host by the mosquito *Aedes aegypti* that has key roles in DENV infection, replication, and viral transmission (vector competence). Mosquito saliva also plays an important role during DENV transmission. In this study, we detected the presence of sialic acid (Sia) in *Aedes aegypti* tissues, which may have an important role during DENV-vector competence. We also identified genome sequences encoding enzymes involved in Sia pathways. The cDNA for *Aedes aegypti* CMP-Sia synthase (CSAS) was amplified, cloned, and functionally evaluated via the complementation of LEC29.Lec32 CSAS-deficient CHO cells. *Aedes*CSAS-transfected LEC29.Lec32 cells were able to express Sia moieties on the cell surface. Sequences related to *α*-2,6-sialyltransferase were detected in the *Aedes aegypti* genome. Likewise, we identified Sia-*α*-2,6-DENV interactions in different mosquito tissues. In addition, we evaluated the possible role of sialylated molecules in a salivary gland extract during DENV internalization in mammalian cells. The knowledge of early DENV-host interactions could facilitate a better understanding of viral tropism and pathogenesis to allow the development of new strategies for controlling DENV transmission.

## 1. Introduction

Dengue fever (DF) is the most important and rapidly expanding arthropod-borne viral disease in tropical areas. Dengue virus (DENV) infection affects more than 100 million people worldwide each year, and 2.5 billion people live in areas of risk [1]. DF is caused by any of the four antigenically distinct dengue virus serotypes (DENV 1–4), which are transmitted to humans by the hematophagous mosquitoes* Aedes (Ae.) aegypti* and* Ae. albopictus*. The recent increase in DF and dengue hemorrhagic fever/dengue shock syndrome, now known as severe dengue, is associated with the vector's expansion to new geographic areas [2]. Severe dengue is a highly pathogenic disease, so the development of a dengue vaccine is a high priority for protecting people at risk, but no safe vaccine is available at present. Therefore, mosquito control is the primary option for preventing dengue outbreaks [3].* Ae. aegypti* females have a key role in DENV-vector competence, which refers to the vector's permissiveness to infection, replication, and viral transmission [3, 4]. The female mosquito acquires DENV from an infected person during blood feeding. The virus undergoes its first replication cycle in the mosquito midgut, before spreading into the hemocoel and finally infecting the salivary glands (SGs). The transfer of infectious saliva into a human host (during a new blood feeding) is a key event during the DENV transmission cycle [4, 5]. Thus, it is very important to identify the molecules involved in the DENV-SG relationship because mosquito saliva is rich in glycoproteins that participate in different host responses (platelet activation, swelling, itching, and inflammation), as well as the binding and transport of vector-borne pathogens to host tissues, thereby allowing pathogens to infect and evade the host immune response [5]. In an ample range of disease models, including various hosts, mosquito species, and arthropod-borne viruses, mosquito saliva and/or mosquito feeding are associated with a potentiation of the arbovirus (arthropod-borne) infection. Host infection via vector saliva leads to an increase in viral transmission, host susceptibility, disease progression, and mortality [6]. The potential for mosquitoes to influence the course of West Nile virus (WNV) disease was investigated by assessing pathogenesis in the presence or absence of mosquito saliva [6]. Likewise,* in vitro* and* in vivo* models of saliva-mediated enhancement of DENV infectivity have been reported [7], but it is uncertain whether* Aedes* saliva glycosylated molecules contributes to DENV tissue infection. The* Aedes* sialome includes 136 putative secretory proteins, which could modify host responses [8]. During DENV-vector infection, the main genes upregulated in* Ae. aegypti* are related to carbohydrate expression [9], but the roles of glycans in vector competence are currently unknown. In addition, it is known that certain glycosidases affect the binding of DENV to mammalian (green monkey kidney and Vero) and mosquito (C636 and AP61) cell surfaces [10]. Previously, it was reported that *β*-glucosidase, sialidase, and heparinase reduce DENV attachment to mammalian cells but not to insect cells [10], and the inability of sialidase to affect DENV binding to insect cells is associated with a lack of mosquito sialyltransferase (ST), which is capable of transferring sialic acid (Sia) residues to mosquito glycoproteins [11]. Moreover, the occurrence of Sia in mosquito tissues is also unknown. However, the genetic and biochemical capacity for sialylation in* Drosophila melanogaster* supports a hypothesis that insect sialylation is a specialized and developmentally regulated process in insects [12–16]. This process is involved in the regulation of neural transmission in the nervous system of* D. melanogaster* [17, 18]. It is well known that sialylated glycoproteins modulate many important biological processes, including cellular and molecular recognition, subcellular and cellular trafficking, intercellular adhesion, and signaling and microbial attachment, among others [19]. In the present study, we detected the presence of a functional cytidine monophosphate- (CMP-)Sia synthase (CSAS) in* Ae. aegypti*, and we also demonstrated that DENV recognizes *α*-2,6-linked Sia structures on the surface of mosquito tissues, which may play key roles during early DENV-vector interactions. Furthermore, we found that DENV is capable of interacting with secretory Sia-glycoproteins, which may be involved in successful DENV-host tissue transmission. To our knowledge, these are the first demonstrations of the functional expression of an* Aedes* CSAS and the presence of Sia moieties in mosquito tissues, which may have important biological consequences for DENV-vector competence. Knowledge of specific early DENV-mosquito interactions could facilitate a better understanding of viral tropism and pathogenesis to allow the development of new effective strategies for the control of DENV transmission, as well as the improvement of antiviral agents and vaccines.

## 2. Materials and Methods

### 2.1. DENV Propagation and Titration

DENV New Guinea C strain serotype 2 (DENV-2, kindly donated by Dr. Duane Gubler, CDC Fort Collins, CO, USA) was propagated in C6/36 cells, which were grown at 28°C in supplemented minimal essential medium (MEM). Confluent monolayers were infected for 2 h at a multiplicity of infection (MOI) of 1 and incubated for 5–7 days at 28°C in a 5% CO_2_ atmosphere until cytopathic effects were observed, before titrating in a lytic plaque assay using LLC-MK2 cells, as described previously [20]. The virus titer was expressed as plaque-forming units (pfu) per milliliter.

### 2.2. *Ae. aegypti* Maintenance, Salivary Glands, Midgut Isolation, and Tissue Extracts

Female* Ae. aegypti* mosquitoes were cultured in an insectarium at the Center for Infectious Disease Research (CISEI-INSP), Mexico. The SGs and midguts of female mosquitoes (at least three days old and fed only with water) were dissected using a microneedle, placed in sterile tubes in groups of 20 pairs with 20 *μ*L of phosphate-buffered saline (PBS) and kept at −75°C. The tissues were lysed during five freeze-thaw cycles using liquid nitrogen and sonicated (ultrasonic 8849-00; Cole-Parmer, IL, USA) for 10 min before centrifugation at 3500 rpm to obtain tissue extracts. The protein concentration was determined using a micro-BCA (bicinchoninic acid) assay (Pierce, USA) at 562 nm with a spectrophotometer (Multiskan Ascent 354, Thermo Labsystem UK).

### 2.3. *Ae. aegypti* Saliva Collection

*Ae. aegypti* saliva was collected as described by Almeras et al. [21], with a small number of modifications. Female mosquitoes were sedated for 1 min at 4°C, and the proboscis of each mosquito was placed in a plastic pipette tip containing mineral oil. After 1 h salivation at room temperature (RT), the liquid was collected from the tip, and the saliva from 20 mosquitoes was pooled, before centrifugation at 10,000 rpm. The protein concentration was estimated using a micro-BCA assay.

### 2.4. Carbohydrate Determination in* Ae. aegypti* Salivary Glands

The salivary glands of female* Ae. aegypti* mosquitoes were dissected as described above, and the SG monosaccharides were analyzed according to Kamerling et al. [22] by GC/MS as trimethylsilyl methyl glycosides (by the Structural and Functional Glycobiology Unit of the University of Sciences and Technologies of Lille, France). Briefly, dry samples were methanolized in methanol/HCl 0.5 N, N-reacetylated, and trimethylsilylated in a mixture of N,O-Bis(trimethylsilyl)trifluoroacetamide and pyridine (1 : 1), before injection into a gas chromatograph with a BPX70 12 m × 0.22 mm diameter column (Chrompack).

### 2.5. Identification of Sia in* Ae. aegypti* Midguts by High-Performance Liquid Chromatography (HPLC)

Midguts were homogenized in water, lyophilized, and incubated in 1 mL 0.1 M TFA at 80°C for 2 h. The samples were centrifuged at 5000 rpm for 15 min, and two volumes of cold ethanol were added to the supernatant. To obtain exact analytical data and to avoid false-positive results, the lyophilized Sias were dried, resuspended in 100 *μ*L of water, and passed successively through 50 × 2 (200 × 400 mesh) and 50 × 8 (25 × 50 mesh) Dowex (100 *μ*L) anion exchange columns (Bio-Rad, Marnes-la-Coquette, France). This sequential cation and anion exchange chromatography process was described in detail in a previous study [23]. The columns were eluted with three volumes of water. The total volume was dried, diluted in one volume of 0.01 M trifluoroacetic acid (TFA), and analyzed by HPLC using a Hewlett-Packard model 1100 liquid chromatography system (Palo Alto, USA), as follows. In the HPLC analysis, Sia was derivatized using 1,2-diamino-4,5-methylenedioxybenzene, according to Hara et al. [24], and separated isocratically in a C-18 reverse phase Sep-PaK HPLC column (250 × 4.6 mm, 5 *μ*m; Vydac, Hesperia, CA, USA) using a solvent mixture of acetonitrile/methanol/water (7 : 9 : 84), followed by identification based on the elution positions of standard Neu5Ac derivatives.

### 2.6. Lectin Histochemistry of* Ae. aegypti* SGs and Midguts

*Ae. aegypti* SGs and midguts were placed on slides and fixed, and the tissues were then blocked with 2% bovine serum albumin (BSA) for 30 min at RT, washed with PBS for 5 min, and immersed in PBS-Triton X-100 (0.2%) for 10 min. Next, they were washed with PBS-Ca^2+^ (1 mM) for 10 min and incubated with different biotin-conjugated lectins, that is,* Maackia amurensis* lectin (MAA),* Sambucus nigra* agglutinin (SNA), or* Lens culinaris* hemagglutinin (LCH) (EY Laboratories, Inc, USA) at 1 : 100 dilutions for 2 h at 37°C. The slides were washed with PBS for 10 min and incubated in the dark with ExtrAvidin-fluorescein isothiocyanate (FITC; Zymed Inc., USA) at 1 : 60. The tissues were then rinsed with PBS-Ca^2+^ (1 mM) for 5 min and with deionized water for 5 min. Finally, the samples were mounted with Vectashield 4′,6-diamidino-2-phenylindole (DAPI; Vectashield, Vector Laboratories, CA, USA) and visualized using a Leica DM fluorescence microscope (DCF-300FX digital camera; Leica Microsystems Digital Imaging, Germany). To evaluate SNA-specific binding, mosquito SGs and different* D. melanogaster* tissues fixed on slides were pretreated with 0.5 IU* Clostridium perfringens* sialidase (Roche Diagnostics, Germany) for 30 min at RT. This sialidase was preincubated with casein and resorufin-labeled according to Twining [25] to prevent protease activity. Samples were incubated in the dark with biotinylated SNA lectin (1 : 100) and streptavidin-FITC (1 : 60). The fluorochromes were analyzed in two channels: green for lectins and blue for nuclei. The gut, SGs, and midgut from* D. melanogaster* were dissected, fixed (as described previously [26]), and incubated with SNA lectin or sialidase. Finally, the images were digitized with the Leica IM1000 version 1.20 program (Imagic Bildverarbeitung AG, Glattbrugg, Switzerland).

### 2.7. DENV-Lectin Binding Assays

SGs were fixed on slides and incubated overnight with DENV (10^7^ pfu) at 4°C. The samples were washed three times each for 10 min using PBS and incubated for 2 h at 37°C with the anti-DENV protein-E antibody (dengue type-2 virus MAB8702; Chemicon International, CA, USA) at a dilution of 1 : 300. Next, the samples were washed with PBS for 10 min and incubated for 20 min at RT in the dark with rhodamine-coupled anti-IgG antibody (Zymed Laboratories, Inc., USA) at a dilution of 1 : 3000. In the competition assays, SGs were incubated with lectins before the addition of DENV. To evaluate the possible participation of Sia in DENV-SG interactions, a DENV-SG competition assay was performed where DENV was preincubated for 1 h with soluble 200 mM Sia (N-acetylneuraminic acid; Sigma-Aldrich) or 1 mM fetuin (DIG Glycan Kit; Roche), before adding it to the SG. Images were acquired in three channels: green for lectins, red for anti-DENV, and blue for nuclei.

### 2.8. Trypsin and Sialidase Assays of SGs and Glycoprotein Identification Using a Lectin Blot Assay

SGs were treated with 0.5 IU of* C. perfringens* sialidase (Roche Applied Science, USA) for 30 min or with 0.075% trypsin (Sigma-Aldrich, Inc, USA) for 5, 15, or 30 min, before the glands were fixed and incubated with DENV. The SGs were incubated with SNA, MAA, or LCH lectins. Finally, images were obtained, as described earlier.

### 2.9. SG Glycoprotein Detection by Blot Assay

Glycoproteins in the SG protein extracts were identified by sodium dodecyl sulfate-polyacrylamide gel electrophoresis (SDS-PAGE) with a polyacrylamide gradient of 4–20%, which was then stained to detect all carbohydrates using a Pro-Q Emerald 300 Glycoprotein Gel Stain kit (Molecular Probes, Invitrogen P21855), according to the supplier's protocol. The gel image was captured under a UV transilluminator (Kodak Gel Logic 1550). For the lectin blot assay, proteins were transferred to nitrocellulose membranes (Trans-Blot 162-0112, Bio-Rad), blocked with 1% BSA + 0.2% Tween-20 in PBS, and washed. The membranes were incubated with biotinylated SNA or* Canavalia ensiformis* agglutinin (ConA; EY Laboratories Inc., USA) at a dilution of 1 : 10 for 3 h at RT, followed by streptavidin-horseradish peroxidase conjugate (43-4323; Zymed Laboratories Inc., USA) at a dilution of 1 : 3000 for 1 h at RT. The membranes were then washed with PBS and visualized with luminol (Western Blotting Reagent sc-2048; Santa Cruz Biotechnology, USA). Finally, the membranes were exposed to a film (Kodak).

### 2.10. Virus Overlay Protein Binding Assays (VOPBA)

VOPBA was performed as described by Salas-Benito and del Angel [27]. Briefly, SG protein extracts or salivary proteins were transferred to nitrocellulose membranes, blocked (1% BSA + 0.2% Tween-20 in PBS) for 1 h at RT, washed three times with PBS, and incubated overnight (4°C) with DENV (10^7^ pfu) in 1% BSA in PBS + 1 mM CaCl_2_. The membranes were washed with PBS and incubated for 3.5 h at RT with a monoclonal antibody against DENV protein E (MAB 8702; Chemicon International, CA, USA) at a dilution of 1 : 300. Next, the membranes were washed twice with PBS + 50 mM NaCl and incubated for 1 h at RT with a secondary anti-mouse IgG antibody (1 : 5000) coupled with peroxidase (81-6520; Zymed Laboratories Inc.). Finally, the membranes were washed, treated with luminol, and exposed to film. To evaluate the role of Sia residues in interactions with DENV, the SG protein extracts and saliva were pretreated with 0.5 IU of* C. perfringens* sialidase (Roche) for 1 h before the overlay assay, as described earlier.

### 2.11. DENV Infection of Mammalian Cells in the Presence of* Ae. aegypti* SG Protein Extract

The internalization of DENV in mammalian cells (LLC-MK2 and wild-type Chinese hamster ovary cells CHO) was assessed in the presence or absence of SG extract protein, where DENV was metabolically labeled with [^35^S]-methionine at 37°C for 1 h. Confluent monolayers of mammalian cells were infected with labeled DENV at an MOI of 1 in the presence or absence of SG proteins extracted from 80 SGs, which were pretreated (or untreated) with 0.5 IU of* C. perfringens sialidase* for 1 h at RT. After infection, the medium was removed, and the cells were washed twice with citrate buffer (10 mM citric acid, 0.05% Tween-20, pH 6.0) and PBS to remove any nonspecifically associated virus after the incubation period, thereby avoiding counting virus that was not internalized. Cells were subsequently lysed and fixed on mats filters (Skatron Instruments, UK). The [^35^S]-methionine level was measured using an LS6500 Scintillation Counter (Beckman Coulter, USA).

### 2.12. LC/ESI-MS/MS Analysis

VOPBA protein bands were selected for protein identification by mass spectrometry (MS) analysis. The bands were carefully excised from Coomassie Brilliant Blue-stained gel and prepared for liquid chromatography-electrospray ionization tandem mass spectrometry (LC-MS/MS). Briefly, individual protein bands were destained, reduced, carbamidomethylated, digested with trypsin, and extracted from the gel using a standard in-gel digestion procedure [28]. The volumes of the extracts were reduced by evaporation in a vacuum centrifuge at RT, before adjusting to 20 *μ*L with 1% formic acid. Peptide MS analysis was performed using a 3200 QTRAP System (Applied Biosystems/MDS, USA), which was equipped with a nanoelectrospray source and a nanoflow LC system (1100 Nanoflow Pump; Agilent, Waldbronn, Germany). Mass tuning of the hybrid triple quadrupole linear IT spectrometer was performed using [Glu1]-fibrinopeptide B. Sample digests were injected into a Zorbax 300SB C18 column equilibrated with 2% ACN and 0.1% formic acid and separated using a linear gradient of 2% to 7% CAN with 0.1% formic acid over an 80 min period, at a flow rate of 300 nL min^−1^. The interface heater used for desolvation was held at 150°C, and the spray voltage was 2.4 kV. Spectra were acquired in the automated mode by information-dependent acquisition. Precursor ions were selected in Q1 using the enhanced MS mode. The scan ranges for EMS were set to 400–1500 and 4000 amu s^−1^. Selected ions were subjected to an enhanced resolution scan at a low speed of 250 amu s^−1^ over a narrow (30 amu) mass range, followed by an enhanced product ion scan (MS/MS). The precursor ions were fragmented by collision-activated dissociation in the Q2 collision cell using rolling collision energy. The fragmented ions were captured and mass analyzed in the Q3 linear IT. Database searches (Swiss-Prot, NCBInr, or MSDB) and protein identification were performed using the MASCOT program (http://www.matrixscience.com/) with trypsin plus one missed cleavage and carboxyamidemethylation as a fixed modification and methionine oxidation as a variable modification, using a mass tolerance of 0.5 Da for the precursor MWs and 0.3 Da for the fragment MWs. The criteria used to accept a protein hit as a valid identification were two or more tryptic peptide matches with the protein sequence and at least one peptide with *P* < 0.05.

### 2.13. Analysis of the Protein Glycosylation Sites

The sequence obtained from the MASCOT database was analyzed with Glycomod [29], which is available at http://www.expasy.ch/tools/glycomod/. This program explores the mass values of ions obtained experimentally with MALDI-ToF and their relationships with sequences in the MASCOT database. The search parameters specified N-glycosylated and O-glycosylated proteins, with modifications of oxidized methylation and cysteine-treated iodoacetamide, using a mass tolerance of 0.1 Da.

### 2.14. *Ae. aegypti* RNA Purification

Groups of 25 female mosquitoes were homogenized and sonicated with RNAse-free water. The lysates were passed through a 0.9 mm needle. RNA extraction was performed using a Nucleospin RNA II kit (Macherey-Nagel, Germany), and the RNA quality was evaluated using Agilent RNA Nano 6000 chips (Agilent 2100 Bioanalyzer).

### 2.15. *Ae. aegypti* CSAS and ST Gene Synthesis

BLink and BLAST searches for CSAS and ST genes were performed using the NCBI tBLAST algorithm based on the CSAS (gi∣24667125) and ST (gi∣24762715) sequences of* D. melanogaster*. Putative CSAS (XP 001663017) and ST (XP 001649590) genes were identified in the* Ae. aegypti* genome and confirmed by VectorBase (https://www.vectorbase.org/) as AAEL012868 and AAEL014772, respectively. cDNA synthesis was performed using 200 ng of RNA template (QPCR cDNA kit; Stratagene, USA) with random primers. Five microliters of cDNA was used in a 25 *μ*L PCR reaction, which was amplified with Taq DNA polymerase (Thermo Fisher Scientific) as follows: 95°C for 5 min; 38 cycles at 94°C for 1 min, 50°C for 1 min, 72°C for 1.5 min, and 72°C for 10 min, holding at 4°C. The following primers were used for* Ae.*CSAS gene synthesis: 5′aedsy (5′GTT GAA TTC CAT GCG GCT AGT TTT GAT 3′), 3′aedsy (5′AAT GGT ACC TTA TTC TAC TGT GGA TCC 3′), 5′aedtr (5′CAC AAG CTT ATG TTG CGT GAC CTT TCG 3′), 3′aedetr (5′CTA GGT ACC TCA ACA TCC ACT GTT GCT 3′), 5′Act (5′TGG TTA CTC GTT CAC CA 3′), and 3′Act (5′GGC ATA CAG ATC CTT TCG GA 3′).

The forward primer 5′aedsy included an* EcoRI* site and the first six codons of* Ae*.CSAS. The 3′aedsy primer contained a* KpnI* site and the last six codons of* Ae*.CSAS. The 5′aedtr forward primer contained a* HindIII* site and the first six codons of the hypothetical* Ae. aegypti* ST sequence, and 3′aedtr included a* KpnI* site and the last six codons of the same sequence. The* Ae. aegypti* actin gene was used as a housekeeping control.

### 2.16. *Ae. aegypti* CSAS cDNA Cloning and Sequencing

The CSAS PCR product was cloned using a Topo vector (Invitrogen) and transformed into* Escherichia coli* strain DH5*α*. The cloned cDNA was evaluated by PCR using M13 forward (–20) and reverse primers. The CSAS cDNA was nicked at the* EcoRI* and* KpnI* sites, and subcloned using a p3XFlag-CMV-10 (Sigma-Aldrich) vector. The plasmid sequence was confirmed by PCR using the primers 5′p3 FLAG (5′-GTTGACGCAAATGGGCGGTAG-3′) and 3′p3 FLAG (5′-CTTGCCCCTTGCTCCATACCAC-3′), as follows: 96°C for 5 min; 38 cycles at 96°C for 45 s, 50°C for 45 s, 72°C for 1 min, and 72°C for 10 min, holding at 4°C. The 786 bp CSAS product was sequenced (Genoscreen, Lille, France).

### 2.17. Complementation of CSAS-Deficient Cells with* Ae.*CSAS

Wild-type CHO cells and LEC29.Lec32 cells, which were deficient in CMP-Neu5Ac synthase, were grown in MEM containing 10% FBS in 5% CO_2_ at 37°C. One million LEC29.Lec32 cells were transfected with lipofectamine reagent (Invitrogen) using 5 *μ*g of the p3XFlag-CMV-10 vector with the* Ae*.CSAS insert or the empty vector as a control. Cells were harvested at 36 h posttransfection.* Ae. aegypti* Sia expression was evaluated by FACS analysis. Cells were detached and incubated for 1 h at 4°C with biotin-conjugated MAA, washed, and incubated for 1 h on ice with Alexa Fluor 488 conjugated streptavidin (Invitrogen). Appropriate isotype and secondary antibody controls were used. In the FACS analysis, 10,000 cells were analyzed using a FACSCalibur system (Becton Dickinson, USA).* Ae*.CSAS expression was also evaluated by histochemistry; that is, WT CHO and LEC29.Lec32 cells were grown on slides and transfected as described previously. Cells were incubated with MAA lectin and Alexa Fluor conjugated antibody and stained in parallel with DAPI.

### 2.18. Hemagglutination Assay with DENV

The assay was performed as described by Goldsmith (see [30] and Casals and Brown [31]). DENV was propagated in C6/36 cells, purified by ultracentrifugation (see Methods in the paper), and suspended in borate solution (pH 9). Borate solution was used as the negative control. In a microtiter plate, a series of twofold dilutions of the viral stock was generated, which was followed by the addition of a suspension of chicken erythrocytes (4% in borate solution) and incubation of the samples for 1 h at 4°C. The hemagglutination activity was expressed as a titer defined as the reciprocal of the maximal dilution that gave positive hemagglutination. A parallel assay was performed using the influenza virus.

### 2.19. Sialidase-Treated Erythrocytes

Sialidase-treated erythrocytes were obtained according to Sano and Ogawa [32]. Briefly, native chicken erythrocytes (10%, v/v) were mixed with an equal volume of the incubation buffer (0.1 M acetate buffer containing 1 mM CaCl_2_, pH 5.5) containing sialidase from* Clostridium perfringens* (1 U/mL), which was preincubated with casein and resorufin to prevent protease activity. The sample was incubated at 37°C for 1 h with occasional careful shaking. The cells were washed by centrifugation using cold PBS (pH 7) and stored as a 10% suspension at 4°C until use. The HA assay was carried out as previously described. A parallel assay was performed using the influenza A virus.

### 2.20. Statistical Analysis

Data were expressed as the mean and standard deviation and compared using a Mann-Whitney *U* test with Statistical Analysis Software version 8 (SAS Institute, USA). The significance level was set at *P* < 0.05. To identify the D7 protein in MASCOT, and the score for an MS/MS match was based on the absolute probability (*P*) that the observed match between the experimental data and the database sequence was a random event. We used a probability-based MOWSE score; that is, the reported score was −10 log (*P*), where *P* was the probability that the observed match was a random event, and the protein scores were significant at *P* < 0.05.

## 3. Results

### 3.1. Identification of Sia in* Ae. aegypti* Mosquito Tissues and Genes Involved in the Sia Synthesis Pathway

**Figure 1:**
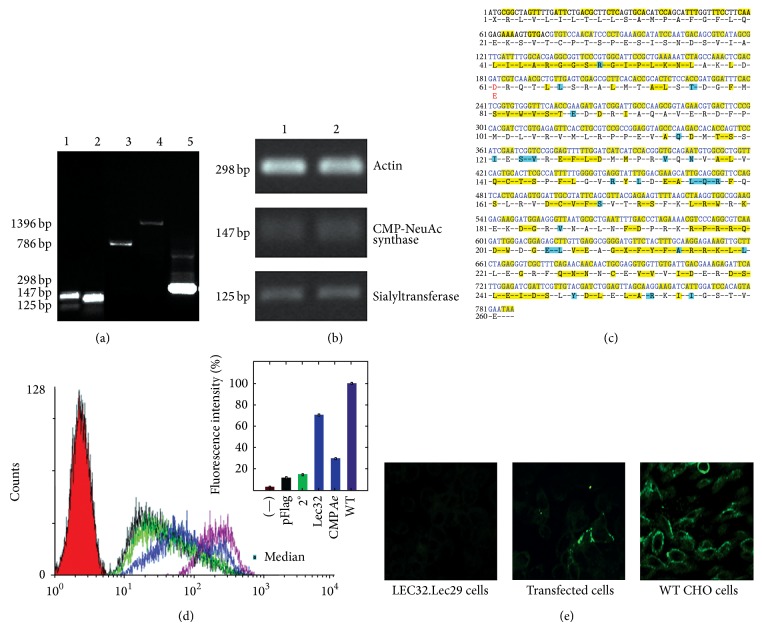
*Ae.*CSAS functional expression evaluation. (a) RT-PCR analysis of* Ae.*CSAS and* Ae.*ST. The figure shows the bands obtained with the internal and external primers of each enzyme using a whole extract of* Ae*.* aegypti* mosquito. Lanes 1-2:* Ae*.CSAS (147 bp) and* Ae*.ST (125 bp) sequences obtained using the internal primers. Lanes 3-4:* Ae.*CSAS (786 bp) and* Ae*.ST (1396 bp) complete sequences obtained with the external primers. Lane 5:* Ae.* actin (298 bp) was used as a housekeeping gene control. (b) RT-PCR analysis of* Ae.*CSAS and ST using total RNA from five pairs of* Ae*.* aegypti* SGs (lane 1) and five midguts (lane 2):* Ae.*CSAS (147 bp);* Ae*.ST (125 bp); and actin control (298 bp). (c) cDNA and aa sequences of* Ae.*CSAS. Identical residues in yellow show multiple alignments with different sequences from other organisms (Figure S1), whereas conserved residues are indicated in blue. (d) Flow cytometry analysis using LEC29.Lec32 untransfected and transfected cells with* Ae*.CSAS, which were incubated with MAA lectin to evaluate Sia expression. Red: isotype control; black: LEC29.Lec32 cells transfected with empty p3XFlag-CMV vector (negative control); green: untransfected cells in the presence of secondary antibody only; blue: LEC29.Lec32 transfected with* Ae.*CSS cDNA; and magenta: wild-type CHO cells (positive control for the expression of *α*-2,3Sia). The bars show the percentage of fluorescence intensity. Approximately 30% of LEC32.Lec29-transfected cells expressed Sia (blue bar) compared with 100% Sia expression in the positive control CHO cells (magenta bar). (e) Affinocytochemistry and confocal microscopy assays using MAA lectin staining to assess Sia expression. Left: LEC29.Lec32-transfected cells with an empty pFlag vector. Center: LEC29.Lec32-transfected cells with the* Ae.*CSAS pFlag vector. Right: wild-type CHO positive control transfected with an empty pFlag vector.

The total carbohydrate composition of the* Ae. aegypti* SG protein extract was determined by gas chromatography, which showed that the most abundant monosaccharide was N-acetylgalactosamine, with an average of 170 *μ*g per 10 salivary glands, followed by mannose (84 *μ*g), N-acetylglucosamine (42 *μ*g), galactose (16 *μ*g), and Sia (Neu5Ac with 7 *μ*g). We also assessed the presence of Sia in midguts using HPLC by referring to the retention times of standard Sia derivatives [33]. Sia was determined at a concentration of 1.4 *μ*g per single midgut. As a consequence of the presence of Sia in different mosquito tissues, we evaluated the possible existence of genes encoding enzymes involved in Sia synthesis pathways. The sialylation process requires the biosynthesis of glycosyl-nucleotide cytidine 5′-monophosphate-N-acetylneuraminic acid (CMP-Neu5Ac) by CSAS and enzymes from the ST family, which transfer Sia to a glycoprotein or glycolipid acceptor substrate. Therefore, using the available genome database of* D. melanogaster*, we searched for the amino acid (aa) sequences of both enzymes, that is, CSAS (gi∣24667125) and D.SialT6 ST (gi∣24762715), and we performed BLAST and BLink analyses of the* Ae. aegypti* genome using the NCBI genome database. We detected hypothetical sequences for both proteins, that is, CSAS (XP 001663017;* Ae*.CSAS) and ST (XP 001649590;* Ae.*ST), in the* Ae. aegypti* genome, which were validated in the VectorBase database. The* Ae. aegypti* ST gene sequence was identified and associated with the ST6Gal, *α*2,6-sialyltransferase (ST6Gal) family, which is closely related to* D. melanogaster* D.ST6 and orthologous to the common ancestral gene that was present before the split of ST6Gal I and ST6Gal II [34]. We used these sequences to generate a complementary DNA (cDNA) that comprised 786 bp for* Ae.*CSAS and another of 1396 bp for* Ae.*ST (Figure 1(a)). Likewise, we obtained* Ae. aegypti* cDNAs for* Ae.*CSAS and* Ae.*ST from the SGs and midguts (Figure 1(b)). The* Ae.*CSAS cDNA was cloned into the p3XFlag-CMV vector. Two clones, that is, C4 synthase and C8 synthase, were sequenced, analyzed, and compared with previously reported CSAS sequences (See Figure S1 in Supplementary Material available online at http://dx.doi.org/10.1155/2015/504187). Both clones contained the start point of an open reading frame for a protein containing 261 aas, with a molecular mass of 29.8 kDa and a theoretical isoelectric point of 6.72. We detected a polymorphism site in the* Ae.*CSAS gene (Figure 1(c)). In clone 4, a point mutation from A (residue 183) to T changed an aspartic acid (D) residue into glutamic acid (E).

### 3.2. Evaluation of* Ae.*CSAS Complementation of CHO Sia-Deficient Cells

To determine the functional activity of* Ae.*CSAS, a p3XFlag-CMV vector containing the* Ae.*CSAS insert was transfected into CHO LEC29.Lec32 cells [35], which were deficient in CSAS expression and did not express sialoglycoconjugates. Sia expression was evaluated by a flow cytometry (FACS) assay using MAA, which recognizes Sia in *α*-2,3-linkages, because CHO cells mainly express *α*-2,3-STs [36]. We observed that* Ae.*CSAS-transfected cells expressed *α*-2,3-linked Sia (Figure 1(d), blue line) at a similar level to the parental CHO cells, which were used as a positive control (Figure 1(d), magenta line). The intensity of fluorescence in the nontransfected CHO LEC29.Lec32 subpopulation was similar to that in the negative control (Figure 1(d), green and black lines). In addition, nearly 30% of the LEC29.Lec32-transfected cells were able to express Sia (Figure 1(d) shows the fluorescence intensity percentages). To confirm the functional activity of* Ae*.CSAS, we tested for the presence of Sia in* Ae.*CSAS-transfected CHO LEC29.Lec32 cells using an affinocytochemical assay with MAA lectin. Sia expression was observed on the cell surface of* Ae.*CSAS-transfected CHO LEC29.Lec32 cells (Figure 1(e)), as shown by the FACS assay. These results demonstrate the functional expression of* Ae*.CSAS in* Ae. aegypti*.

### 3.3. DENV-Sia Interaction in* Ae. aegypti* Tissues

**Figure 2:**
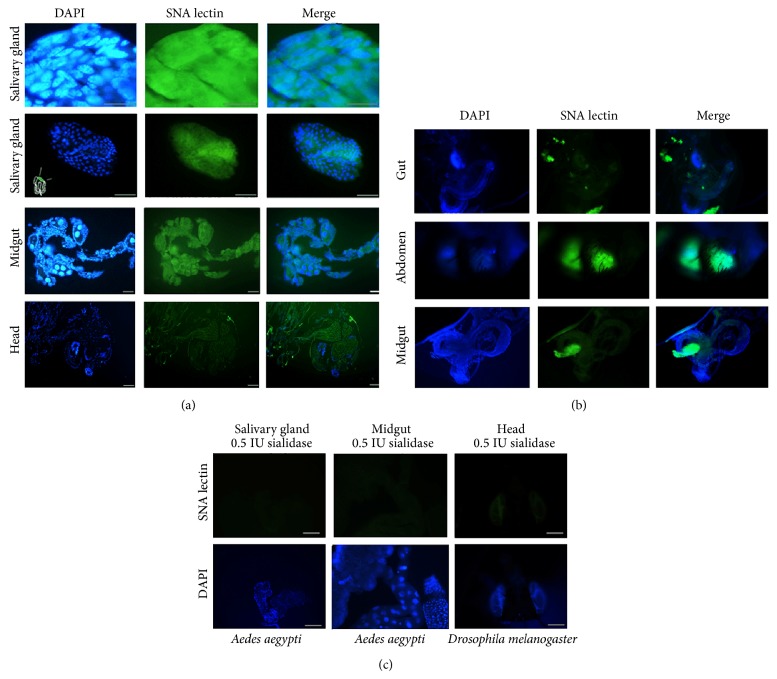
Lectin histochemistry of* Ae. aegypti* tissues. (a) Results of *α*-2,6-linked Sia detection in* Ae. aegypti* SG, midgut, and head incubated with SNA lectin (1 : 100) and stained with FITC. SG, upper panel: 60x microscopic magnification, lower panel: 40x lens. The inner box in the SG-DAPI panel shows the SG region analyzed. To identify Sia, the midgut and head transverse sections were evaluated with SNA lectin (green) (20x magnification). (b) Results for the *α*-2,6-linked Sia positive control in* D. melanogaster* abdomen, gut, and midgut using SNA lectin, which are similar to those for* Ae. aegypti* tissues. (c) SNA staining of mosquito SG and midgut pretreated with 0.5 IU sialidase for 30 min before SNA incubation. The control comprised* D. melanogaster* heads pretreated with sialidase. Blue: nuclei stained with DAPI. Green: (FITC) SNA lectin interaction.

The* Ae. aegypti* ST gene is related to the ST6Gal family [37]; thus, we evaluated gene expression based on the presence of *α*-2,6-Neu5Ac moieties on the surface of mosquito tissues (SG, head, and midguts) using affinocytochemistry and confocal microscopy assays with the lectin SNA, which recognizes Sia in *α*-2,6-linkages. We observed strong SNA staining in the different mosquito samples (Figure 2(a)).* D. melanogaster* tissues were used as the positive control and are well known [15] to express *α*-2,6-linked Neu5Ac moieties (Figure 2(b)). No MAA binding was observed in* Ae. aegypti* tissues, which indicates that* Ae. aegypti* does not express *α*-2,3-ST (similar to* D. melanogaster*, Figure S2). To validate the SNA binding assay, SGs were pretreated with* C. perfringens* sialidase and incubated with SNA lectin. In the absence of sialidase treatment, strong SNA staining was observed in* Ae. aegypti* mosquito and* D. melanogaster* tissues (Figures 2(a) and 2(b)). However, the SNA binding decreased after sialidase treatment of the mosquito and* D. melanogaster* tissues (Figure 2(c)).

**Figure 3:**
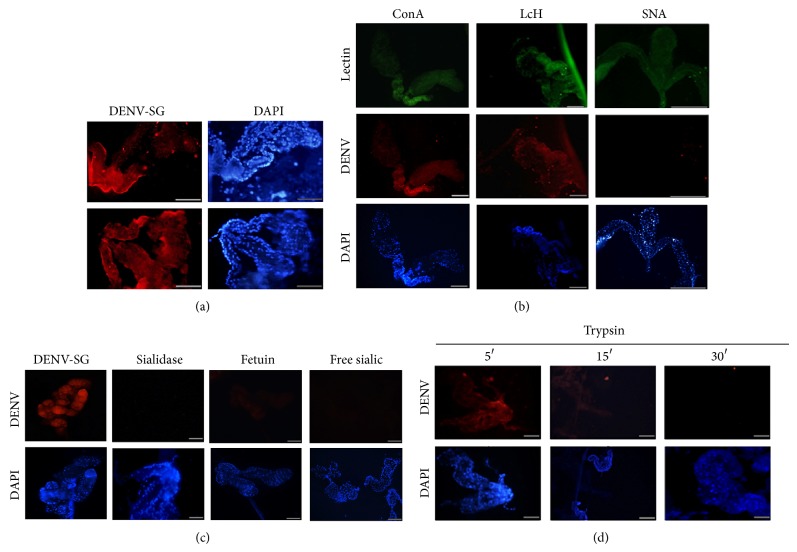
DENV interaction with* Ae. aegypti* SG. (a) DENV interaction with* Ae. aegypti* SGs. SGs from* Ae. aegypti* were incubated with DENV and stained with anti-DENV E antibody and rhodamine-coupled anti-IgG antibody. (b) DENV-SG competence assays using ConA, LCH, and SNA lectins, which were added to SG before incubation with DENV. The interaction with DENV was blocked when DENV was incubated in the presence of lectins that recognized Sia. With LCH and ConA lectins, the magnification = 10x and with SNA lectin = 20x. Scale bar = 10 *μ*m. (c) DENV-SG interaction in the absence or presence of sialidase. SGs were untreated or pretreated with* C. perfringens* sialidase for 30 min before adding DENV. The DENV-SG interactions in the presence of Sia competitors, fetuin (1 mM) and free Sia (200 nM), are also shown, where the DENV-SG interaction was blocked. (d) DENV-SG interaction in SGs pretreated with trypsin for 5, 15, or 30 min before adding DENV. There was a decrease in the DENV-SG interaction after 15 min, and it was lost completely at 30 min. Scale bar = 10 *μ*m. Blue: nuclei stained with DAPI. Red: DENV stained with an antibody against viral protein E and a secondary antibody coupled to rhodamine. Green: (FITC) SNA lectin interaction.

SG is the main tissue where DENV is replicated and amplified in the mosquito before transmission to its vertebrate host; thus, we evaluated the possible role of Sia in DENV-SG interaction. We performed a binding assay with* Ae. aegypti* SG in the presence of different lectins (SNA, LCH, or ConA).  Figure 3(a) shows that there was a positive DENV-SG interaction in the absence of SNA lectin. However, DENV binding decreased when *α*-2,6-Sia residues were blocked with SNA (Figure 3(b)), whereas the blocking of mannose residues with ConA or LCH did not modify the DENV-SG interaction (Figure 3(b); DENV-midgut interaction Figure S3). To confirm the possible role of Sia during DENV-SG binding, SGs were pretreated with* C. perfringens* sialidase at 30 min prior to DENV addition. We observed a large decrease in the DENV-SG interaction when the SGs were pretreated with sialidase (Figure 3(c)). To evaluate the specific role of Sia in DENV-SG binding, we performed a DENV-SG competition assay using free Neu5Ac and sialylated glycoprotein fetuin. We observed that the DENV-SG interaction decreased in the presence of fetuin, and it was lost in the presence of free Neu5Ac (Figure 3(c)), thereby suggesting the involvement of Sia in DENV-SG recognition. SGs were pretreated with trypsin for 5, 15, or 30 min to determine whether the sialylated molecules related to DENV-SG were proteins (Figure 3(d)). The interaction with DENV decreased after 15 min of incubation and it was abolished completely at 30 min. These data suggest the possible participation of sialylated glycoproteins in DENV tissue attachment.

### 3.4. Detection of* Ae. aegypti* SG Glycoproteins by Blot Assays

**Figure 4:**
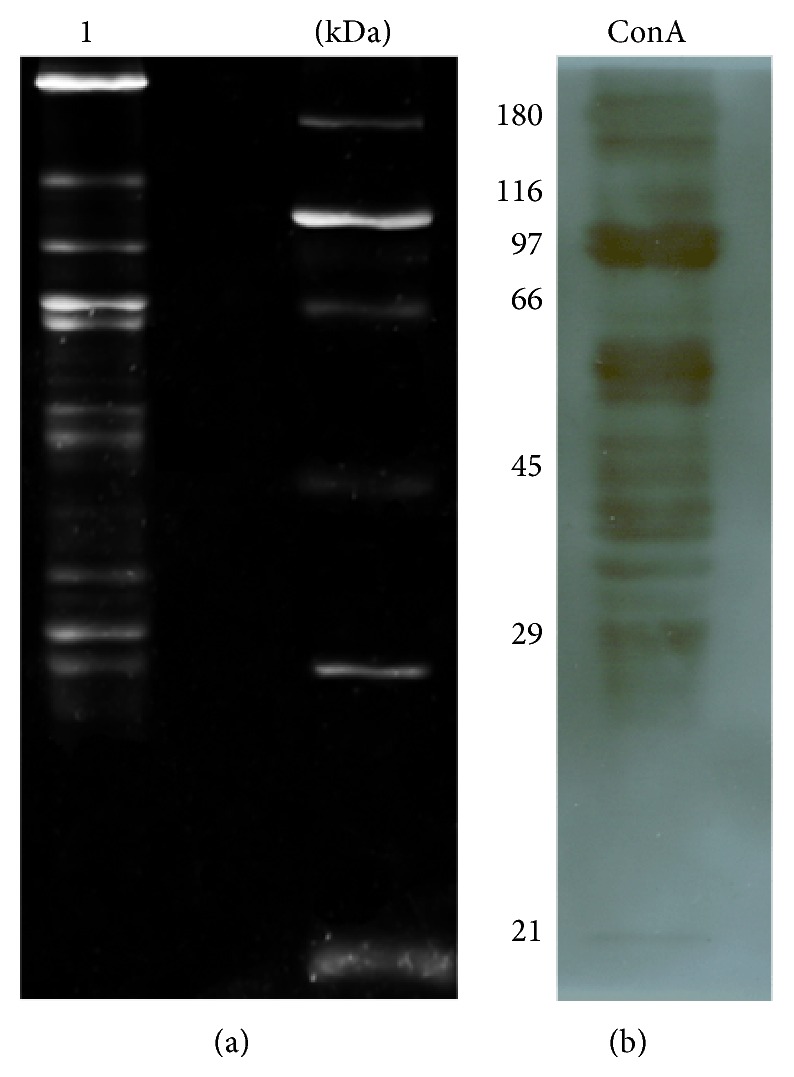
SDS-PAGE assay of the glycoproteins from* Ae. aegypti* SG protein extracts. (a) Total carbohydrates stained with Pro-Q Emerald, where the molecular weights are shown on the right. (b) Western blot assay using ConA lectin, which binds to glycoproteins that contain mannose or glucose residues.

**Figure 5:**
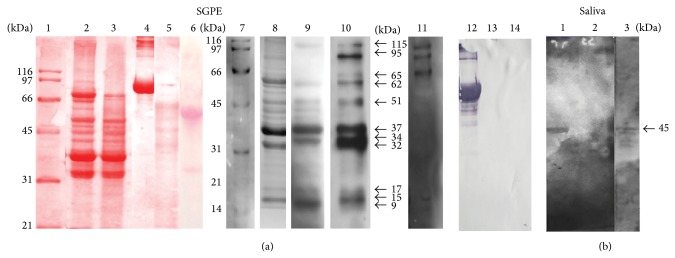
DENV overlay assay with* Ae. aegypti* SG protein extract (SGPE) and* Aedes* saliva in the presence or absence of* C. perfringens* sialidase. (a) DENV-SGPE interactions. Lanes 1–6 show nitrocellulose membranes stained with Ponceau red. Lane 1: MW markers; lane 2: SGPE; lane 3: SGPE pretreated with sialidase; lane 4: fetuin glycoprotein; lane 5: asialofetuin; and lane 6: fetuin pretreated with sialidase. Lanes 7–12 show the blot and overlay assays of SGPE. Lane 7: MW markers; lane 8: SGPE; lane 9: blot of SGPE with SNA lectin; lane 10: DENV overlay with SGPE; lane 11: DENV overlay with SGPE pretreated with sialidase; lane 12: blot of fetuin glycoprotein with SNA lectin; lane 13: blot of asialofetuin with SNA lectin; and lane 14: blot of SNA lectin with fetuin pretreated with sialidase. (b) DENV-saliva interactions. Lane 1: blot of mosquito saliva with SNA lectin; lane 2: DENV overlay with saliva pretreated with sialidase; and lane 3: overlay of DENV-saliva proteins.

To confirm the presence of total sugars in the SG protein extracts from* Ae. aegypti* and to characterize the putative glycoprotein(s) that may recognize DENV, we separated the SG proteins by electrophoresis and stained them to detect any carbohydrates. The SG protein extracts were transferred to nitrocellulose membranes and subjected to a western blot assay. The membrane was also incubated with ConA or SNA lectins (Figures 4(b) and 5(a), lane 9). For the control assay, we used a carbohydrate staining kit (Pro-Q Emerald 300 Glycoprotein Gel Stain Kit, Molecular Probes; Figure 4(a), lane 1), and we observed a range of glycoproteins from 29 kDa to 116 kDa, with more intense bands of 29, 45, and 66 kDa. When we incubated the SG protein extracts proteins with ConA, we observed a glycoprotein of 50–60 kDa, which has not been identified previously with the carbohydrate staining kit. We also observed an increase in the intensity of the band at 97 kDa. Therefore, these proteins could have contained mannose and glucose residues (Figure 4(b)). The interaction with SNA produced several bands that ranged from 10 to 97 kDa (Figure 5(a), lane 9), so these proteins could possess Sia motifs. In agreement, we observed no significant changes when we pretreated the SG protein extracts with sialidase (Figure 5(a), lanes 2 and 3).

### 3.5. Identification of DENV Attachment Glycoproteins in* Ae. aegypti* SGs and Saliva

To identify putative sialylated glycoproteins involved in DENV-SG interactions, different VOPBAs were performed using* Ae. aegypti* SGs and saliva. We observed that DENV interacted with different SG proteins with approximate molecular weights (MWs) of 115, 95, 65, 62, 51, 37, 34, 32, 17, 15, and 9-10 kDa (Figure 5(a), lane 10). The proteins with MWs from 65 to 9 kDa were also observed in the samples detected with SNA lectin (Figure 5(a), lane 9). To test the possible participation of Sia in DENV-mosquito protein interactions, we performed a parallel VOBPA assay, where we pretreated protein extracts from the SGs or saliva with sialidase. Interestingly, DENV protein binding was partially or totally abolished in both cases (Figure 5(a), lane 11; Figure 5(b), lane 2). It was also interesting that the SG proteins of 95 and 65 kDa, which did not interact with SNA lectin (Figure 5(a), lane 9), were not affected in the VOBPA pretreated with sialidase (Figure 5(a), lane 11). In the saliva-DENV binding assay, we observed a protein with a MW of 45 kDa (Figure 5(b), lane 3), which was also present in the samples with SNA lectin (Figure 5(b), lane 1), but it was eliminated when we used sialidase in the VOPBA (Figure 5(b), lane 2). Thus, we propose that the DENV-mosquito SG interaction is at least partially dependent on the presence of Sia residues. We used the sialylated glycoprotein fetuin as a positive control for SNA lectin (Figure 5(a), lanes 4 and 12), whereas asialofetuin (Figure 5(a), lanes 5 and 13) and fetuin pretreated with* C. perfringens* sialidase were used as the negative controls (Figure 5(a), lanes 6 and 14).

### 3.6. Identification of* Ae. aegypti* SG and Saliva Glycoproteins by LC/ESI-MS/MS

The different DENV-SG and DENV-saliva binding proteins observed in the VOPBAs were identified by LC/ESI-MS/MS analysis. The identities of the SG and saliva proteins are shown in Table 1. The DENV-SG binding proteins were as follows: (1)* Aedes* apyrase, which is a protein that hydrolyzes ATP and ADP to adenosine, thereby inhibiting ADP-dependent platelet aggregation; (2)* Aedes* salivary serpin, which is an anticoagulant molecule that inhibits coagulation factor Xa [38]; and (3) the* Aedes* long form of the D7 salivary protein. D7 is the most abundant subfamily of salivary proteins, and they are classified as odorant pheromone-binding proteins, although they also function as scavengers of biogenic amines [39]. They also include (4) the* Aedes* 30-kDa SG allergen. Glycosylated proteins are associated with allergies [40]. Another one of the DENV-SG binding proteins is (5) the* Aedes* putative 34 kDa secreted salivary protein, which is distributed widely in mosquito saliva. The protein product of the 34 kDa family had significant matches with cytoskeletal proteins such as actin and myosin, mainly because of the presence of a repeated charged aa [41]. Another one of the DENV-SG binding proteins is (6) the* Aedes* 14.5 kDa salivary protein, which has an unknown function. Another one of the DENV-SG binding proteins is (7) the* Aedes* short form of the D7 salivary protein, which can bind biogenic amines such as serotonin, histamine, and epinephrine [41]. The sequestration of biogenic amines during mosquito feeding is an important function that inhibits platelet aggregation, vasoconstriction, and inflammation. Another one of the DENV-SG binding proteins is (8) the* Aedes* putative C-type lectin. In mammalian cells, two membrane C-type lectins, DC-SIGN and L-SIGN, interact with DENV via high-mannose glycans on viral glycoproteins [42], while another C-type lectin, the mannose receptor, interacts with the DENV envelope protein, which may enhance viral attachment to phagocytes [43]. It has also been demonstrated that the* Ae. aegypti* C-type lectin recognizes West Nile virus* in vivo* and* in vitro* during cell infection [44]. Another one of the DENV-SG binding proteins is (9) the* Aedes* beta subunit protein translocation complex. Silencing of the* Drosophila* and human ortholog gene (Sec61) of the beta subunit protein significantly reduces DENV infections in the S2 cell line and HuH-7 cells [45]. The ion masses and the sequences of the SG proteins involved in DENV interactions were evaluated using Glycomod to determine whether the proteins were putative glycoproteins with Sia motifs (Supplementary File 1).

The 45-kDa saliva protein that interacts with specific lectins for Sia as well as with DENV is similar to the peptide ion mass of the protein NCBI: gi∣157113327 [Vectorbase: AAEL006417-RA], which is a putative molecule in the D7 family of* Ae. aegypti*. It had a 35% match in its primary sequence, with a score of 178 and an expected value of 6.4^−13^ (*P* < 0.05). Based on the analysis of the sequence of the putative D7 protein from* Ae. aegypti*, we identified a transmembrane region between aa residues 7 (phenylalanine) and 24 (leucine) from the amino terminus (Figure S4). Therefore, it can be considered as a membrane protein, although it has been suggested that members of this family of proteins are secreted in the salivary glands of various mosquitoes [46, 47]. We also noted that the D7 protein contains potential N-glycosylation sites, specifically in the region of aas 278–284 (Supplementary File 1). There were two possible combinations of carbohydrates involving Sia: the first was combined with hexose, and the second with N-acetylglucosamine or N-acetylgalactosamine. We evaluated the potential Sia-glycosylation sites some of which have little differences in terms of the ionic masses obtained with MALDI-ToF (experimental mass), the theoretical mass of the glycopeptides, and the carbohydrate mass. In addition, we only considered differences of <0.05 Da, and three peptide regions in the D7 protein had these characteristics. Between residues 35–39, there were two possible combinations of O-linked glycosylation via the hydroxyl groups of serine and threonine: the first combination involved the binding of Sia to two molecules of N-acetylglucosamine or N-acetylgalactosamine; and the second involved a combination with hexose, NeuAc, and ketodeoxynonulosonic acid. The second peptide with the potential to be O-glycosylated was in the region of aas 285–290, where a threonine residue could be linked to pentose, N-acetylglucosamine, or N-acetylgalactosamine, and Sia residues. Finally, there was a serine residue in the region of aas 311–316, where the difference between the experimental mass and theoretical mass was only 0.019 Da. Therefore, it is possible that a Sia residue linked to a deoxyhexose occurs in this region.

### 3.7. DENV Infection of Mammalian Cells in the Presence of* Ae. aegypti* SG Protein Extracts

**Figure 6:**
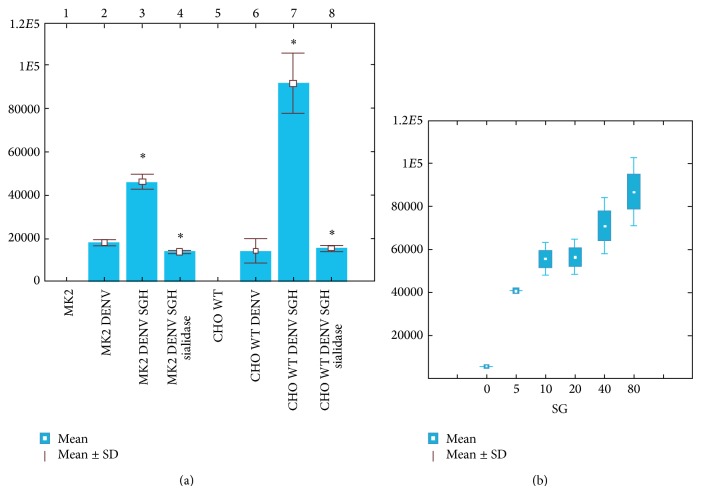
DENV-mammalian cells internalization assay. (a) DENV internalization by LLC-MK2 and CHO cells. The plot shows the internalization of [^35^S]-methionine-radiolabeled DENV by LLC-MK2 and CHO cells in the absence (lanes 2 and 6) and presence (lanes 3 and 7) of* Ae. aegypti* SG protein extract (SGH) and in the presence of SGH pretreated with sialidase before DENV incubation (lanes 4 and 8). DENV was mixed with SGH (from 80 SGs), which was pretreated or untreated with sialidase, before infecting mammalian cells with the DENV-SGH mixture. In the plot, the *y*-axis shows the counts per min of internalized DENV, ^*^ *P* < 0.05. (b) DENV internalization by CHO cells in the presence of different amounts of SGH. The plot shows that DENV internalization was enhanced by the presence of the protein extract from five SGs, which was dose dependent.

It is known that* Ae. aegypti* saliva enhances West Nile and Cache Valley virus infections, but it is unknown whether* Aedes* saliva can modulate DENV infections [6]. Based on our detection of interactions between DENV and salivary glycoproteins, we evaluated the possible participation of the* Ae. aegypti* SG protein extract in the modulation of DENV infection in different mammalian cell lines (LLCMK2 and CHO WT) using a DENV internalization assay, in the presence or absence of SG extracts. We found that DENV infection was enhanced in the presence of SG extract in both mammalian cell lines (Figure 6(a)). CHO cells appeared to be more permissive (fourfold enhancement; Figure 6(a), lane 7) than LLCMK2 (twofold enhancement; Figure 6(a), lane 3). We pretreated the SG protein extract with sialidase before the internalization assay to evaluate the possible participation of Sia during DENV cell internalization, and we observed the effect on DENV internalization, which was reduced in sialidase-pretreated samples (Figure 6(a), lanes 4 and 8). The internalization of DENV in CHO cells in the presence of different amounts of SG protein extract was dose dependent, as shown in Figure 5(b). These results support a general hypothesis that molecules in mosquito saliva and secretory SG proteins can potentiate pathogen-host transmission and that Sia residues play a role during DENV internalization in mammalian cells.

## 4. Discussion

Sialylation is a biologically important modification of glycoconjugates, which is observed mainly in the deuterostome lineage. However, the occurrence of this process in protostomes is less clear [19]. Using the available* Ae. aegypti* genome database, we identified two putative genes encoding enzymes (*Ae.*CSAS and* Ae.*ST) implicated in the* Ae. aegypti* sialylation pathway. The cDNA of* Ae.*CSAS was amplified, cloned, and functionally evaluated by the complementation of CSAS-deficient LEC29.Lec32 CHO cells. Sia moieties were present at the cell surface in* Ae.*CSAS-transfected CHO LEC29.Lec32 cells. The identification of a functional Sia synthase in* Ae. aegypti* indicates that* Aedes* mosquitoes have the biosynthetic capacity for endogenous Sia production. Our data are consistent with previous studies [12–16] of the expression of a functional* D. melanogaster* CSAS and the presence of *α*-2,6-linked Sia moieties in* D. melanogaster*. Sia is distributed widely in nature at the nonreducing termini of glycoproteins, glycolipids, or secreted glycoconjugates, and it may be attached to different acceptors via *α*-2,3, *α*-2,6, or *α*-2,8-linkages, which are determined by the specificity of different STs [48]. In this study, we demonstrated the presence of* Ae. aegypti* ST cDNAs in different* Ae. aegypti* tissues (Figures 1(a) and 1(b)) and observed the presence of *α*-2,6-linked Sia moieties (in a lectin binding assay) at the tissue level. These data are consistent with a report where it was shown that arthropods STs, including* Ae. aegypti* ST, are associated with the ST6Gal ST family, which is orthologous to the common ancestral gene that was present before the split of ST6Gal I and ST6Gal II in vertebrates [34].

To our knowledge, this is the first report of the presence of Sia glycans in* Ae. aegypti* tissues. The type of Sia linkage also plays a key role in the specific recognition of different viruses, because *α*-2,3- or *α*-2,6-specificity could define the cell and host tropism [49]. For example, human influenza A virus hemagglutinin binds primarily to Neu5Ac*α*2-6Gal structures, whereas avian influenza virus binds specifically to Neu5Ac*α*2-3Gal [50]. This specificity limits the cell tropism and viral host range significantly. The participation of *α*-2,6-Sia structures during early DENV-vector interactions may have key roles in DENV infection, host tropism, and viral pathogenesis.

It was reported that* Anopheles* salivary glands contain several glycoconjugates in the surface, which are critical for recognition of different pathogens [51–53]. Perrone et al. [54] suggested that the salivary gland carbohydrate complexity reflects the functional diversity of this tissue. By lectin-binding assay, the authors detected the presence of *α*-D-mannose, *α*-D-N-acetyl-galactosamine, *β*-D-gal-(1,3) N-acetyl-galactosamine, *β*-D-galactose, N-acetyl-galactosamine, *α*-L-fucose, and *β*-N acetyl-glucosamine. Likewise, different oligosaccharide structures such as Man3GlcNAc2, Man3 (Fuc) 1-2GlcNAc2 were detected [55]. Recently, Francischetti et al. [56] demonstrated the presence of sulfated glycans in the salivary gland of* Anopheles gambiae*. Because of the glycan complexity in the vector salivary glands and in order to ensure that sialic acid detection in* Aedes aegypti* mosquito tissues was specific, the role of Sia in DENV-SG binding was evaluated by a DENV-SG competition assay using free sialic acid and also the sialylated glycoprotein fetuin. We observed that the DENV-SG interaction decreased in the presence of fetuin, and it was lost in the presence of free sialic acid (Figure 3(c) and supplementary Figure 5(B)–5(E)). In the same way, we observed in a hemagglutination assay of dengue virus with sialylated red blood cell (chicken erythrocytes) an inhibitory effect in presence of free sialic acid. Moreover, the Sia participation in DENV-sialic acid interaction was confirmed by the loss of hemmagglutination activity in the presence of desialylated erythrocytes (Supplementary Table 1).

DENV cellular infection is a multistep process that involves different molecules, some of them present in* Aedes *tissues like the laminin receptor, the tubulin like protein, HSP90 protein, unknown proteins of Mw: 35, 40–45, 48, 74, and 80 KDa and several detergent-soluble proteins of salivary glands with Mw: 35–80 KDa [57]. However, neither evaluated the possible participation of Sia glycoconjugates [58, 59], and the occurrence and participation of Sia in interactions among mosquito tissues and DENV have not been considered previously.

However, the participation of Sia in* Plasmodium gallinaceum* ookinetes-midgut interactions has been documented previously. Zieler et al. [60] reported that the chemical modification of the midguts from* Ae. aegypti* mosquito with a periodate concentration of <1 mmol inhibit the adhesion of ookinetes in the midguts, and they also found that free N-acetylneuraminic acid competed for ookinete binding to midguts. Interestingly, Barreau et al. [61] found that wheat-germ agglutinin (WGA) lectin, which binds residues of N-acetylglucosamine, blocks the interaction between* Plasmodium gallinaceum* sporozoites and the surface of* Ae. aegypti* SGs. WGA is a* Triticum vulgaris* lectin that specifically recognizes N-acetylglucosamine residues, but it also has regions that interact with Sia residues. These reports suggest the possible participation (and presence) of sialic acid in the interactions between mosquito tissue and* Plasmodium*. Colpitts et al. [9] reported that Sia residues are important for the recognition of DENV in mammalian (Vero and LLC-MK2) cells, and a large number of DENV binding molecules are known [58, 62–66]. However, there have been no evaluations of the possible role during DENV-vector-host transmission.

In the present study, we found that a sialylated saliva glycoprotein (45 kDa Figure 5(b) lane 1) of* Ae. aegypti* forms complexes with DENV. This protein belongs to the D7 proteins family and is secreted in the saliva [21]; thus, it could be implicated in DENV host transmission. The modulation of DENV infection in different mammalian cells by* Aedes* salivary extracts and the observation that desialylated salivary proteins decrease DENV internalization highlight the key roles played by sialylated molecules during DENV vector-host interactions. Several studies have demonstrated the effects of arthropod saliva on vertebrate responses in a wide range of disease models using various hosts, arboviruses, and mosquito species [5, 6, 67, 68]. In all cases, an increase in virus transmission, modification of host susceptibility, or disease progression were observed. The enhancement of infection as a result of SG extracts is attributed to the modulation of host immune response, reduction of T-lymphocytes, and antiviral activity [69]. In the current study, we detected enhanced DENV internalization in presence of* Aedes* SG extracts, but the virus internalization decreased when the salivary proteins were pretreated with sialidase. In agreement with our results, Surasombatpattana et al. [70, 71] observed enhanced DENV infection of human keratinocytes in the presence of SG extracts. Recently, Conway et al. [7] reported that the* Aedes aegypti* saliva serine protease activity enhances dissemination of DENV into the mammalian host, although the role of Sia was not considered. Identification of molecules that mediate infectivity enhancement will allow for the production of vector-based vaccines and therapeutics that will target arthropod saliva components and interfere with viral transmission, as is exemplified by antimaxadilan (MAX) and anti-SP15 vaccines [72, 73]. These data may represent a general property for other vector-borne pathogens as is the case of* Plasmodium*. The knowledge of early DENV-host interactions could lead to a better understanding of viral tropism and pathogenesis and provide information for the development of new strategies for the control of DENV transmission.

To our knowledge, this is the first report of the participation of Sia structures during early interactions between DENV and* Ae. aegypti* mosquito tissues.

## Supplementary Material

Supplementary Figure 1: CLUSTALW analysis of *Aedes aegypti* CSAS.
Supplementary Figure 2: MAA Lectin in the midguts of *Aedes aegypti* and the SGs of *Aedes aegypti* and Drosophila melanogaster.
Supplementary Figure 3: DENV interaction with *Aedes aegypti* midgut.
Supplementary Figure 4: Mass analysis of ions obtained with MALDI–ToF and the equivalent peptide sequences.
Supplementary Figure 5: SNA staining in *Aedes aegypti* mosquito and Drosophila melanogaster tissues in the presence or absence of C. perfringens sialidase.
Supplementary Table 1. Hemagglutinating activity of DENV.

## Figures and Tables

**Table 1:** Identification of DENV-2 binding proteins from *Ae. aegypti* SGs and saliva proteins.

Protein name	NCBI accession number	MW (kDa)		Number of matched peptides	Score	Sequence coverage (%)
		Gel	Database			
**SG protein extract**						
Apyrase [*Aedes aegypti*]	gi∣556272	62.820	62.691	14	404	19%
Salivary serpin [*Aedes aegypti*]	gi∣94469320	51.617	47.765	49	712	60%
D7 protein, putative [*Aedes aegypti*]	gi∣157113327	37.200	38.603	46	862	44%
Long form D7Bclu1 salivary protein [*Aedes aegypti*]	gi∣16225992	37.200	38.579	5	86	15%
D7 protein [*Aedes aegypti*]	gi∣159557	37.200	37.005	3	51	9%
Putative 34 kDa secreted protein [*Aedes aegypti*]	gi∣18568296	34.833	36.154	18	547	33%
Putative 34 kDa family secreted salivary protein [*Aedes aegypti*]	gi∣94468336	34.833	35.698	20	533	33%
30 kDa salivary gland allergen Aed a 3 [*Aedes aegypti*]	gi∣2114497	32.628	27.130	37	479	55%
Allergen, putative [*Aedes aegypti*]	gi∣157133926	32.628	29.529	13	216	31%
Short form D7Cclu23 salivary protein [*Aedes aegypti*]	gi∣16225995	16.947	17.676	10	150	24%
Putative salivary C-type lectin [*Aedes aegypti*]	gi∣94468370	16.947	17.202	5	104	17%
Putative 14.5 kDa salivary protein [*Aedes aegypti*]	gi∣94468650	14.862	17.039	6	117	40%
Protein translocation complex beta subunit, putative [*Aedes aegypti*]	gi∣157138304	9.397	10.329	2	75	24%

**Saliva**						
D7 Protein putative [*Aedes aegypti*]	gi∣157113327	45.23	39.173	18	178	35%

Proteins were identified by LC/ESI-MS/MS analysis after gel trypsin digestion. The table shows the protein name, the NCBI accession number, the theoretical (database) and observed (gel) MWs, the number of peptide sequences matched in the MASCOT database, the corresponding percentage sequence coverage, and the MASCOT score. The criteria used for accepting a protein as a valid identification were two or more tryptic peptide matches with the protein sequence and at least one peptide with *P* < 0.05.

## References

[1] World Health Organization (WHO) (2009). *Dengue Guidelines for Diagnosis, Treatment, Prevention and Control*.

[2] Gubler D. J. (2004). The changing epidemiology of yellow fever and dengue, 1900 to 2003: full circle?. *Comparative Immunology, Microbiology & Infectious Diseases*.

[3] Halstead S. B. (2008). Dengue virus-mosquito interactions. *Annual Review of Entomology*.

[4] Anderson J. R., Rico-Hesse R. (2006). Aedes aegypti vectorial capacity is determined by the infecting genotype of dengue virus. *The American Journal of Tropical Medicine and Hygiene*.

[5] Titus R. G., Bishop J. V., Mejia J. S. (2006). The immunomodulatory factors of arthropod saliva and the potential for these factors to serve as vaccine targets to prevent pathogen transmission. *Parasite Immunology*.

[6] Schneider B. S., Higgs S. (2008). The enhancement of arbovirus transmission and disease by mosquito saliva is associated with modulation of the host immune response. *Transactions of the Royal Society of Tropical Medicine and Hygiene*.

[7] Conway M. J., Watson A. M., Colpitts T. M. (2014). Mosquito saliva serine protease enhances dissemination of dengue virus into the mammalian host. *Journal of Virology*.

[8] Thangamani S., Wikel S. K. (2009). Differential expression of *Aedes aegypti* salivary transcriptome upon blood feeding. *Parasites and Vectors*.

[9] Colpitts T. M., Cox J., Vanlandingham D. L. (2011). Alterations in the aedes aegypti transcriptome during infection with west nile, dengue and yellow fever viruses. *PLoS Pathogens*.

[10] Thaisomboonsuk B. K., Clayson E. T., Pantuwatana S., Vaughn D. W., Endy T. P. (2005). Characterization of dengue-2 virus binding to surfaces of mammalian and insect cells. *The American Journal of Tropical Medicine and Hygiene*.

[11] Stollar V., Schlesinger R. W. (1980). Togaviruses in cultured arthropod cells. *The Togaviruses: Biology, Structure, Replication*.

[12] Roth J., Kempf A., Reuter G., Schauer R., Gehring W. J. (1992). Occurrence of sialic acids in *Drosophila melanogaster*. *Science*.

[13] Kim K., Lawrence S. M., Park J. (2002). Expression of a functional Drosophila melanogaster N-acetylneuraminic acid (Neu5Ac) phosphate synthase gene: evidence for endogenous sialic acid biosynthetic ability in insects. *Glycobiology*.

[14] Koles K., Irvine K. D., Panin V. M. (2004). Functional characterization of *Drosophila* sialyltransferase. *The Journal of Biological Chemistry*.

[15] Aoki K., Perlman M., Lim J.-M., Cantu R., Wells L., Tiemeyer M. (2007). Dynamic developmental elaboration of N-linked glycan complexity in the *Drosophila melanogaster* embryo. *The Journal of Biological Chemistry*.

[16] Viswanathan K., Tomiya N., Park J. (2006). Expression of a functional *Drosophila melanogaster* CMP-sialic acid synthetase: Differential localization of the drosophila and human enzymes. *The Journal of Biological Chemistry*.

[17] Repnikova E., Koles K., Nakamura M. (2010). Sialyltransferase regulates nervous system function in Drosophila. *The Journal of Neuroscience*.

[18] Islam R., Nakamura M., Scott H. (2013). The role of *Drosophila cytidine* monophosphate-sialic acid synthetase in the nervous system. *Journal of Neuroscience*.

[19] Varki A. (2007). Glycan-based interactions involving vertebrate sialic-acid-recognizing proteins. *Nature*.

[20] Cabello-Gutiérrez C., Manjarrez-Zavala M. E., Huerta-Zepeda A. (2009). Modification of the cytoprotective protein C pathway during Dengue virus infection of human endothelial vascular cells. *Thrombosis and Haemostasis*.

[21] Almeras L., Fontaine A., Belghazi M. (2010). Salivary gland protein repertoire from *Aedes aegypti* mosquitoes. *Vector-Borne and Zoonotic Diseases*.

[22] Kamerling J. P., Gerwig G. J., Vliegenthart J. F., Clamp J. R. (1975). Characterization by gas-liquid chromatography-mass spectrometry and proton-magnetic-resonance spectroscopy of pertrimethylsilyl methyl glycosides obtained in the methanolysis of glycoproteins and glycopeptides. *Biochemical Journal*.

[23] Reuter G., Schauer R. (1994). Determination of sialic acids. *Methods in Enzymology*.

[24] Hara S., Yamaguchi M., Takemori Y., Furuhata K., Ogura H., Nakamura M. (1989). Determination of mono-*O*-acetylated *N*-acetylneuraminic acids in human and rat sera by fluorometric high-performance liquid chromatography. *Analytical Biochemistry*.

[25] Twining S. S. (1984). Fluorescein isothiocyanate-labeled casein assay for proteolytic enzymes. *Analytical Biochemistry*.

[26] Tian E., Zhang L., Hagen K. G. T. (2013). Fluorescent lectin staining of drosophila embryos and tissues to detect the spatial distribution of glycans during development. *Methods in Molecular Biology*.

[27] Salas-Benito J. S., del Angel R. M. (1997). Identification of two surface proteins from C6/36 cells that bind dengue type 4 virus. *Journal of Virology*.

[28] Kinter M., Sherman N. E. (2000). The preparation of protein digests for mass spectrometric sequencing experiments. *Protein Sequencing and Identification Using Tandem Mass Spectrometry*.

[29] Cooper C. A., Gasteiger E., Packer N. H. (2001). GlycoMod—a software tool for determining glycosylation compositions from mass spectrometric data. *Proteomics*.

[30] Goldsmith R. S. (1966). Assay of dengue virus in monkey kidney cells by detection of hemagglutinin in the culture medium. *American Journal of Epidemiology*.

[31] Casals J., Brown L. V. (1954). Hemagglutination with arthropod-borne viruses. *The Journal of Experimental Medicine*.

[32] Sano K., Ogawa H. (2014). Hemmagglutination (inhibition) assay. *Lectin Methods and Protocols, Methods in Molecular Biology*.

[33] Schauer R., Srinivasan G. V., Coddeville B., Zanetta J.-P., Guérardel Y. (2009). Low incidence of *N*-glycolylneuraminic acid in birds and reptiles and its absence in the platypus. *Carbohydrate Research*.

[34] Koles K., Repnikova E., Pavlova G., Korochkin L. I., Panin V. M. (2009). Sialylation in protostomes: a perspective from *Drosophila* genetics and biochemistry. *Glycoconjugate Journal*.

[35] Potvin B., Raju T. S., Stanley P. (1995). lec32 is a new mutation in Chinese hamster ovary cells that essentially abrogates CMP-N-acetylneuraminic acid synthetase activity. *The Journal of Biological Chemistry*.

[36] Stanley P., Shantha Raju T., Bhaumik M. (1996). CHO cells provide access to novel N-glycans and developmentally regulated glycosyltransferases. *Glycobiology*.

[37] Petit D., Mir A.-M., Petit J.-M. (2010). Molecular phylogeny and functional genomics of *β*-galactoside *α*2,6-sialyltransferases that explain ubiquitous expression of st6gal1 gene in amniotes. *The Journal of Biological Chemistry*.

[38] Stark K. R., James A. A. (1998). Isolation and characterization of the gene encoding a novel factor Xa-directed anticoagulant from the yellow fever mosquito, *Aedes aegypti*. *The Journal of Biological Chemistry*.

[39] Calvo E., Mans B. J., Andersen J. F., Ribeiro J. M. C. (2006). Function and evolution of a mosquito salivary protein family. *The Journal of Biological Chemistry*.

[40] Simons F. E. R., Peng Z. (2001). Mosquito allergy: recombinant mosquito salivary antigens for new diagnostic tests. *International Archives of Allergy and Immunology*.

[41] Ribeiro J. M. C., Arcà B., Lombardo F. (2007). An annotated catalogue of salivary gland transcripts in the adult female mosquito *Aedes aegypti*. *BMC Genomics*.

[42] Klimstra W. B., Nangle E. M., Smith M. S., Yurochko A. D., Ryman K. D. (2003). DC-SIGN and L-SIGN can act as attachment receptors for alphaviruses and distinguish between mosquito cell and mammalian cell-derived viruses. *Journal of Virology*.

[43] Miller J. L., de Wet B. J. M., Martinez-Pomares L. (2008). The mannose receptor mediates dengue virus infection of macrophages. *PLoS Pathogens*.

[44] Cheng G., Cox J., Wang P. (2010). A C-type lectin collaborates with a CD45 phosphatase homologue to facilitate West Nile virus infection of mosquitoes. *Cell*.

[45] Sessions O. M., Barrows N. J., Souza-Neto J. A. (2009). Discovery of insect and human dengue virus host factors. *Nature*.

[46] Arcà B., Lombardo F., de Lara Capurro M. (1999). Trapping cDNAs encoding secreted proteins from the salivary glands of the malaria vector Anopheles gambiae. *Proceedings of the National Academy of Sciences of the United States of America*.

[47] Arcà B., Lombardo F., Lanfrancotti A. (2002). A cluster of four D7-related genes is expressed in the salivary glands of the African malaria vector *Anopheles gambiae*. *Insect Molecular Biology*.

[48] Harduin-Lapers A. (2010). Comprehensive analysis of sialyltransferases in vertebrate genomes. *Glycobiology Insights*.

[49] Lehmann F., Tiralongo E., Tiralongo J. (2006). Sialic acid-specific lectins: occurrence, specificity and function. *Cellular and Molecular Life Sciences*.

[50] Suzuki Y., Ito T., Suzuki T. (2000). Sialic acid species as a determinant of the host range of influenza A viruses. *Journal of Virology*.

[51] Brennan J. D. G., Kent M., Dhar R., Fujioka H., Kumar N. (2000). Anopheles gambiae salivary gland proteins as putative targets for blocking transmission of malaria parasites. *Proceedings of the National Academy of Sciences of the United States of America*.

[52] James A. A. (2003). Blocking malaria parasite invasion of mosquito salivary glands. *The Journal of Experimental Biology*.

[53] Ghosh A. K., Jacobs-Lorena M. (2009). Plasmodium sporozoite invasion of the mosquito salivary gland. *Current Opinion in Microbiology*.

[54] Perrone J. B., DeMaio J., Spielman A. (1986). Regions of mosquito salivary glands distinguished by surface lectin-binding characteristics. *Insect Biochemistry*.

[55] Li J. S., Vavricka C. J., Christensen B. M., Li J. (2007). Proteomic analysis of N-glycosylation in mosquito dopachrome conversion enzyme. *Proteomics*.

[56] Francischetti I. M., Ma D., Andersen J. F., Ribeiro J. M. (2014). Evidence for a lectin specific for sulfated glycansin the salivary gland of the malaria vector, Anopheles gambiae. *PLoS ONE*.

[57] Hidari K. I. P. J., Suzuki T. (2011). Dengue virus receptor. *Tropical Medicine and Health*.

[58] Cabrera-Hernandez A., Smith D. R. (2005). Mammalian dengue virus receptors. *Dengue Bulletin*.

[59] Smith D. R. (2012). An update on mosquito cell expressed dengue virus receptor proteins. *Insect Molecular Biology*.

[60] Zieler H., Nawrocki J. P., Shahabuddin M. (1999). Plasmodium gallinaceum ookinetes adhere specifically to the midgut epithelium of Aedes aegypti by interaction with a carbohydrate ligand. *The Journal of Experimental Biology*.

[61] Barreau C., Touray M., Pimenta P. F., Miller L. H., Vernick K. D. (1995). *Plasmodium gallinaceum*: sporozoite invasion of *Aedes aegypti* salivary glands in inhibited by anti-gland antibodies and by lectins. *Experimental Parasitology*.

[62] Mendoza M. Y., Salas-Benito J. S., Lanz-Mendoza H., Hernández-Martinez S., del Angel R. M. (2002). A putative receptor for dengue virus in mosquito tissues: localization of a 45-KDA glycoprotein. *The American Journal of Tropical Medicine and Hygiene*.

[63] Mercado-Curiel R. F., Esquinca-Avilés H. A., Tovar R., Díaz-Badillo Á., Camacho-Nuez M., de Lourdes Muñoz M. (2006). The four serotypes of dengue recognize the same putative receptors in *Aedes aegypti* midgut and *Ae. albopictus* cells. *BMC Microbiology*.

[64] Mercado-Curiel R. F., Black W. C., Mũoz M. D. L. (2008). A dengue receptor as possible genetic marker of vector competence in *Aedes aegypti*. *BMC Microbiology*.

[65] Cao-Lormeau V.-M. (2009). Dengue viruses binding proteins from *Aedes aegypti* and *Aedes polynesiensis* salivary glands. *Virology Journal*.

[66] Muñoz M. D. L., Limón-Camacho G., Tovar R., Diaz-Badillo A., Mendoza-Hernández G., Black W. C. (2013). Proteomic identification of dengue virus binding proteins in *Aedes aegypti* mosquitoes and *Aedes albopictus* cells. *BioMed Research International*.

[67] Titus R. G., Ribeiro J. M. C. (1988). Salivary gland lysates from the sand fly *Lutzomyia longipalpis* enhance leishmania infectivity. *Science*.

[68] Styer L. M., Kent K. A., Albright R. G., Bennett C. J., Kramer L. D., Bernard K. A. (2007). Mosquitoes inoculate high doses of West Nile virus as they probe and feed on live hosts. *PLoS Pathogens*.

[69] Schneider B. S., Soong L., Coffey L. L., Stevenson H. L., McGee C. E., Higgs S. (2010). Aedes aegypti saliva alters leukocyte recruitment and cytokine signaling by antigen-presenting cells during West Nile virus infection. *PLoS ONE*.

[70] Surasombatpattana P., Patramool S., Luplertlop N., Yssel H., Missé D. (2012). Aedes aegypti saliva enhances dengue virus infection of human keratinocytes by suppressing innate immune responses. *The Journal of Investigative Dermatology*.

[71] Surasombatpattana P., Ekchariyawat P., Hamel R. (2014). *Aedes aegypti* saliva contains a prominent 34-kDa protein that strongly enhances dengue virus replication in human keratinocytes. *Journal of Investigative Dermatology*.

[72] Morris R. V., Shoemaker C. B., David J. R., Lanzaro G. C., Titus R. G. (2001). Sandfly maxadilan exacerbates infection with *Leishmania major* and vaccinating against it protects against *L. major* infection. *Journal of Immunology*.

[73] Valenzuela J. G., Belkaid Y., Garfield M. K. (2001). Toward a defined anti-Leishmania vaccine targeting vector antigens: characterization of a protective salivary protein. *Journal of Experimental Medicine*.

